# Recognition of sentinel lymph nodes in patients with papillary thyroid cancer by nano-carbon and methylene blue

**DOI:** 10.12669/pjms.336.13940

**Published:** 2017

**Authors:** Fangzhou Liu, Yan Zhu, Yichuan Qian, Jia Zhang, Yu Zhang, Yuan Zhang

**Affiliations:** 1 Fangzhou Liu, Department of Head and Neck Surgery, Jiangsu Cancer Hospital Affiliated to Nanjing Medical University, Nanjing 210009, P. R. China; 2 Yan Zhu, Department of Pathology, The First Affiliated Hospital of Nanjing Medical University, Nanjing 210029, P. R. China; 3 Yichuan Qian, Department of Head and Neck Surgery, Jiangsu Cancer Hospital Affiliated to anjing Medical University, Nanjing 210009, P. R. China; 4 Jia Zhang, PET-CT Department, Jiangsu Cancer Hospital Affiliated to Nanjing Medical University, Nanjing 210009, P. R. China; 5 Yu Zhang, School of Biological Sciences and Medical Engineering, Southeast University, Nanjing 211189, P. R. China; 6 Yuan Zhang, Department of Head and Neck Surgery, Jiangsu Cancer Hospital Affiliated to Nanjing Medical University, Nanjing 210009, P. R. China

**Keywords:** Papillary thyroid cancer, Sentinel lymph node, Nano-carbon, Methylene blue

## Abstract

**Objective:**

To compare the accuracy and feasibility of methylene blue and nano-carbon in clinical tracing of sentinel lymph nodes (SLNs) in patients with papillary thyroid cancer (PTC).

**Methods:**

Ninety-six PTC patients were selected and randomly divided into a methylene blue group and a nano-carbon group (n=48). During surgery, tracer agent was injected around the tumor, and SLNs were resected and subjected to frozen pathological examination. The results were compared with those of routine pathological examination after surgery.

**Results:**

Latent lymph node metastasis (level VI and lateral neck) was detected in both groups, with neck distribution of SLNs. There was no significant difference in the detection rate or accuracy of SLNs between two groups (P>0.05). The incorrect resection rate of parathyroid gland and incidence of temporary hypoparathyroidism in the methylene blue group were significantly higher than those of the nano-carbon group (t=4.137, P<0.05).

**Conclusions:**

The state of PTC lymph nodes can be well evaluated by SLN biopsy using both methylene blue and nano-carbon as tracers, but using nano-carbon has a lower incidence rate of parathyroid injury, with great clinical prospects accordingly.

## INTRODUCTION

Papillary thyroid cancer (PTC), despite with the lowest degree of malignancy, accounts for about 85% of thyroid tumors, but its incidence has been increasing annually. It has become the fifth susceptible malignancy for females, which mostly endangers children or young women, with an uncertain age of onset.^1^,^2^ Sentinel lymph nodes (SLNs) first drain lymph of primary cancer in local tissues, biopsy for which can accurately predict the state of regional lymph nodes.^3^

Recently, researchers have endeavored to carry out SLN biopsy for melanoma, breast cancer, penile cancer and colon cancer, providing a scientific basis for determining the surgical range.^4^ However, SLNs of thyroid cancer have seldom been studied. Nano-carbon, as a novel lymph tracer, has rarely been applied to thyroid cancer therapy. At present, SLN biopsy is mainly traced by staining method, radionuclide method and combination method. For staining method, methylene blue and nano-carbon are mainly employed, which are cheap and expensive respectively.

In this study, 96 PTC patients were selected as subjects to compare the detection rate, accuracy and postoperative complications of methylene blue and nano-carbon in the tracing of SLN biopsy.

## METHODS

Ninety-six PTC patients admitted in our hospital between April 2016 and February 2017 were selected, and subjected to neck ultrasound, chest X-ray and thyroid function examinations before surgery. There were 19 males and 77 females aged between 45 and 69 years old, with a mean of (51.98 ± 3.1). The patients were randomly divided into a methylene blue group and a nano-carbon group (n=48). The two groups had similar age and gender ratio (P>0.05). This prospective study has been approved by the ethics committee of our hospital, and written informed consent has been obtained from all patients. Sample size (n) of each group = 2(mse/D2) × (Q + μβ), where mse is the mean square of error and D is the inter-group difference. In this study, mse=50u/dl and D=4u/u. Referring to a known table, Q=4.2 and μβ=1.645, so n=2(50/42)×(4.2+1.645)≈37. Therefore, over 37 cases should be included in each group. Considering the actual situation simultaneously, we included 96 cases in total. Sampling was performed using the routine operational steps of frozen pathological analysis.

### Inclusion criteria

Patients who were diagnosed as PTC and without cervical lymph node metastasis through preoperative ultrasound examination were included.

### Exclusion criteria

Patients with benign thyroid lesions or other types of thyroid cancer diagnosed by intraoperative frozen pathological examination, a history of neck or thyroid surgery or neck radiotherapy, or distant systemic metastasis were excluded.

### Determination criteria

According to the clinical evaluation criteria for neck lymph nodes proposed by Kouvaraki et al.,^5^ the following requirements should all be met: Clinical palpation found no enlarged lymph nodes or the maximum diameter was <20 mm, with soft texture; B ultrasound or CT examination disclosed no enlarged lymph nodes or the maximum diameter was <10 mm, or the maximum diameter was 10-20 mm, with a >2 aspect ratio though. The shape was regular, and the cortex and medulla had clear boundary. There was no sand-like small calcification, central liquefaction necrosis, peripheral enhancement or disappearance of fatty space near lymph nodes; patients who lacked imaging data were diagnosed based on palpation.

### Surgical methods

An anterior cervical low-collar incision (2 cm above the suprasternal notch) was made under general anesthesia. The anterior cervical flap and central neck line were separated to expose the thyroid surface and to confirm the location of primary tumor. At the 3-, 6-, 9- and 12-o’clock positions about 1-2 mm around the tumor, 0.2-0.8 mL of 10 g/L methylene blue was injected for the methylene blue group and 1 mL of nano-carbon suspension was injected for the nano-carbon group using a 1 mL syringe. Thyroid gland was kept in the original anatomical position. The lobes of affected thyroid gland were removed 2-5 minutes after oppression on the puncture point with a sterile gauze.

There were three cases of lateral cervical lymph node metastasis in two groups each. The surgical approach was bilateral total thyroid lobectomy + level VI lymph node dissection of affected side + levels II-V functional neck dissection of affected side. All remaining cases were given total thyroid lobectomy of affected side + isthmus resection + level VI lymph node dissection of affected side.

### Evaluation criteria for SLN biopsy

Using the results of routine postoperative pathological examination as golden standards, the evaluation criteria for SLN biopsy were as follows: 1) Positive: SLN metastasis; 2) negative: no metastasis of SLN or non-SLN. The following indices were calculated: Detection rate = (number of cases with detected SLNs/number of all enrolled cases) × 100%; sensitivity = (number of positive SLN cases/ number of cases with cervical lymph node metastasis) × 100%; accuracy = (number of positive cases + number of negative cases)/number of recognized SLNs × 100%.

### Pathological examination and monitoring of postoperative complications

All surgically resected samples (including thyroid gland lobes, SLNs, level VI lymph nodes of affected side, and levels II-V cervical lymph nodes of affected side) were subjected to routine pathological examination. The incorrectly resected parathyroid glands suggested by intraoperative frozen and routine postoperative pathological examinations were counted, and parathyroid hormone and blood calcium levels were detected on the 1st, 3rd and 30th days after surgery, respectively. Whether the patients had tetany, numbness and other symptoms of hypocalcemia was observed. In case of hypocalcemia, calcium was supplemented through intravenous infusion for two days and then through oral administration. Subsequently, the patients were followed up for one year. If there was still hypoparathyroidism and hypocalcemia after drug withdrawal, it was permanent hypoparathyroidism. If not, it was temporary hypoparathyroidism. Whether there were other postoperative complications (incision bleeding, infection, effusion, hoarseness, irritating cough while drinking water, chylous fistula and toxic or allergic reactions caused by injecting tracers) was also observed.

### Statistical analysis

All data were analyzed by SPSS 22.0. The categorical data were expressed as x ± s, and inter-group comparisons were performed by the t test. The numerical data were compared by fourfold tables using the Chi-square test or continuity corrected Chi-square test. Areas under the ROC curves were analyzed to compare the accuracy of two methods. The effects of number of detected SLNs on accuracy were assessed by ROC curves. P<0.05 was considered statistically significant.

## RESULTS

### SLN detection rate and distribution

The SLN detection rate of the methylene blue group (89.6%, 43/48) was similar to that of the nano-carbon group (95.8%, 46/48) (P>0.05) (Table-I). The rate of latent lymph node metastasis was 46.88% (45/96), with 40.63% (39/96) in Level VI and 6.25% (6/96) in lateral neck. In the methylene blue group, 1~4 SLNs were detected in each case, (1.93 ± 0.69) on average. In the nano-carbon group, 1~5 SLNs were detected in each case, (2.60 ± 0.89) on average. There was a significant difference (t=-3.83, P<0.01). The two groups had similar neck distributions of SLNs (P>0.05).

**Table-I:** SLN detection rate and distribution (case).

*Group*	*Tumor size*		*TNM stage*		*Number of SLNs*	*SLN in Level VI*	*Positive SLN in Level VI*	*Non-positive SLN in Level VI*	*SLN in lateral neck*	*Positive SLN in lateral neck*	*Non-positive SLN in lateral neck*

	*≤1 cm*	*>1 cm*	*T1, T2*	*T3, T4*							
Methylene blue	29	19	32	16	43	38	17	3	6	4	0
Nano-carbon	27	21	34	14	46	39	18	1	7	3	0
*t*	1.362	1.023	1.035	1.910	1.266	1.654	1.781	1.354	1.874	1.456	2.059
*P*	>0.05	>0.05	>0.05	>0.05	>0.05	>0.05	>0.05	>0.05	>0.05	>0.05	>0.05

There was no significant difference in the sensitivity or accuracy of SLNs between the two groups (P>0.05) (Table-II).

**Table-II:** SLN biopsy-related results (% (case/case)).

*Group*	*Sensitivity*	*Accuracy*
Methylene blue	93.75 (45/48)	85.42 (41/48)
Nano-carbon	95.83 (46/48)	93.75 (45/48)
*t*	1.579	1.542
*P*	>0.05	>0.05

### Incorrect resection rate of parathyroid gland and temporary hypoparathyroidism

The incorrect resection rate of parathyroid gland in the methylene blue group (16.67%, 8/48) was significantly higher than that of the nano-carbon group (2.08%, 1/48) (t=4.137, P<0.05). Besides, 6 cases (12.50%) in the methylene blue group suffered from temporary hypoparathyroidism, but none in the nano-carbon group did so, between which the difference was significant (t=4.137, P<0.05).

### Other complications

Neither group suffered from surgical site bleeding, infection, effusion, postoperative hoarseness, water drinking-induced irritating cough, chylous fistula, or tracer agent injection-induced toxicity or allergic reaction.

## DISCUSSION

SLN, as the first stop of tumor metastasis, is the node for drainage of primary cancers in regional lymphoid tissues.^6^ The metastasis is helpful to determine tumor stage, prognosis and treatment regimen.^7^ There have been many reports since SLN biopsy was first applied in thyroid cancer surgery in 1998.^8^-^11^ Up to now, the SLN tracer methods include radionuclide method, staining method and their combination. In the radionuclide method and the combination method, thyroid SLN is close to tumor, so they can be greatly disrupted by thyroid tumor and thyroid radionuclide, needing special instruments and causing radioactive contamination. Therefore, the application is restricted. Currently, the staining method generally uses methylene blue and nano-carbon suspension, with the SLN detection rates of 66.70%-94.7% and 91.67%-97.20% respectively.^12^

In this study, the SLN detection rates of methylene blue and nano-carbon groups were 89.6% and 95.8% respectively, without significant differences. Although (2.60 ± 0.89) SLNs were detected in the nano-carbon group, which significantly exceeded that of the methylene blue group (1.93 ± 0.69), the number of each group could not effectively evaluate the accuracy of positive SLNs. Using postoperative routine pathological results as the golden standard, the two groups had similar SLN accuracies. Accordingly, though nano-carbon increased the number of detected SLNs per patient compared with methylene blue, it failed to elevate the detection rate or accuracy (P<0.05). However, the postoperative rate of parathyroid injury (including mistaken incision rate and postoperative temporary hypoparathyroidism) of the nano-carbon group was significantly lower than that of the methylene blue group. Therefore, in the PTC operation of CN0, the nano-carbon method better protected parathyroid glands and reduced the rate of postoperative parathyroid injury compared with the methylene blue method. Probably, when SLN was tracked by nano-carbon, lymph nodes were black, and parathyroid gland was not stained; but when SLN was tracked by methylene blue, both lymph nodes and parathyroid were stained blue, which could hardly be identified.^13^

PTC is the most common pathological type of thyroid cancer, accounting for 60% to 80% of all thyroid cancers, of which 27% to 90% exist occult lymphatic metastasis.^14^ Lymph node metastasis is an important factor in PTC recurrence, but its impact on prognosis remains elusive.^15^ As the lymph node metastasis rate of PTC central region (i.e. Level VI) is high, routine elimination of regional lymph nodes can reduce local regional recurrence rate. In addition, about 20% of PTC may be transferred to lymph nodes outside Level VI at the first stop.^16^,^17^ Thus, it is necessary to accurately assess the cervical lymph nodes of CN0 patients, as well as to reduce the complications caused by local recurrence and re-operation due to metastasis. SLN biopsy can identify whether there is regional lymph node metastasis in tumors, which has been widely applied in breast cancer and melanoma.^18^

Herein, the occult lymph node metastasis rate was 46.88%, of which 40.63% underwent Level VI lymph node metastasis and 6.25% had lateral cervical lymph node metastasis. Therefore, it was necessary to carry out SLN biopsy for PTC patients. Both methylene blue and nano-carbon methods had high accuracies for SLN detection, and had similar SLN distributions in the neck. Hence, both methods can be used in PTC SLN biopsy to evaluate latent lymph node metastasis. Except for parathyroid injury rate, no other complications occurred in either group, suggesting that both methods were safe and reliable for PTC SLN biopsy.

## CONCLUSION

In summary, both methylene blue and nano-carbon functioned well in evaluating lymph node states in SLN biopsy, with similar detection rates and accuracies. Nevertheless, compared with the methylene blue method, the nano-carbon method better protected parathyroid gland sand reduced the incidence of postoperative hypoparathyroidism. Thus, the nano-carbon method shows more promising prospects for clinical use.

### Authors’ Contributions

***Fangzhou Liu, Yan Zhu, Yu Zhang & Yuan Zhang:*** Study design and manuscript preparation.

***Yichuan Qian and Jia Zhang:*** Data collection and analysis.
